# Epithelial Ovarian Carcinoma Associated with Metastases to Central Nervous System: Two Case Reports

**DOI:** 10.1155/2018/4301247

**Published:** 2018-10-02

**Authors:** Yasuyuki Kawagoe, Tetsuo Nakayama, Satoshi Matuzawa, Kazuko Fukushima, Junji Onishi, Yuichiro Sato, Kimihiro Nagai, Hiroshi Sameshima

**Affiliations:** ^1^Department of Obstetrics and Gynecology, Faculty of Medicine, University of Miyazaki, 5200 Kihara, Kiyotake, Miyazaki 889-1692, Japan; ^2^Department of Diagnostic Pathology, University of Miyazaki Hospital, Faculty of Medicine, University of Miyazaki, 5200 Kihara, Kiyotake, Miyazaki 889-1692, Japan; ^3^Department of Palliative Care, Miyazaki Medical Association Hospital, 738-1 Funado, Shinbeppu, Miyazaki City, Miyazaki 889-0834, Japan

## Abstract

We experienced two rare cases of metastases to the central nervous system (cerebral and leptomeningeal metastases) from primary epithelial ovarian carcinoma. The first case was a 55-year-old woman who developed carcinomatous meningitis while on chemotherapy for ovarian cancer stage IIIC. Cytological analysis confirmed carcinomatous cells of ovarian origin in the cerebrospinal fluid. Magnetic resonance imaging demonstrated abnormal hyperintensity in the cerebral sulci on fluid attenuated inversion recovery (FLAIR) sequence with enhanced gadolinium indicating leptomeningeal metastases. Her consciousness rapidly declined and she died 42 days after diagnosis. The second case was a 63-year-old woman who underwent surgery for ovarian cancer and who was diagnosed as stage IA. Thirty-eight months after surgery, she developed weakness of the left hand and headaches. A CT scan revealed metastases to the right cerebrum and she was treated with surgical resection followed by radiotherapy. Five months after resection, she developed ileus caused by multiple relapses in the pelvis. Despite chemotherapy, her performance status declined and she died nine months after the resection. Both cases were rare because the first case was isolated leptomeningeal metastases, and the second case was confirmed relapse site in the cerebrum due to neurological symptoms despite her early clinical stage.

## 1. Introduction

In Japan, 9,804 cases of ovarian carcinoma and 4,758 disease-related deaths annually were reported in 2009 [1]. Almost half of the cases were higher than stage III at initial diagnosis, which usually results in a poor prognosis. Metastases from primary epithelial ovarian carcinoma usually occurs in the abdomen, lungs, and lymph nodes, but metastasis to the central nervous system (CNS) is rare. Advancements in the therapy of ovarian cancer have improved the patient response rate and prolonged the survival period. However, it has also increased the incidence of CNS metastases, which usually develop after long-term treatment. In this report, we describe two types of metastases to the CNS, cerebral and leptomeningeal metastases, from epithelial ovarian carcinoma.

## 2. Case Presentation

**Figure 1:**
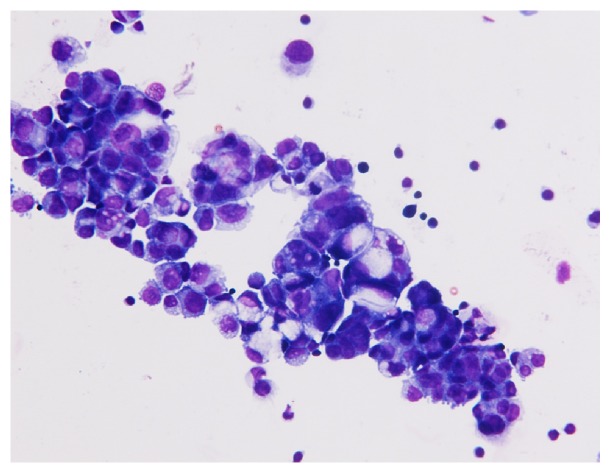
Numerous atypical epithelial clusters were present in the CSF. These atypical cells have eccentric large hyperchromatic nuclei and scant cytoplasm. Some cells show vacuolar changes.

**Figure 2:**
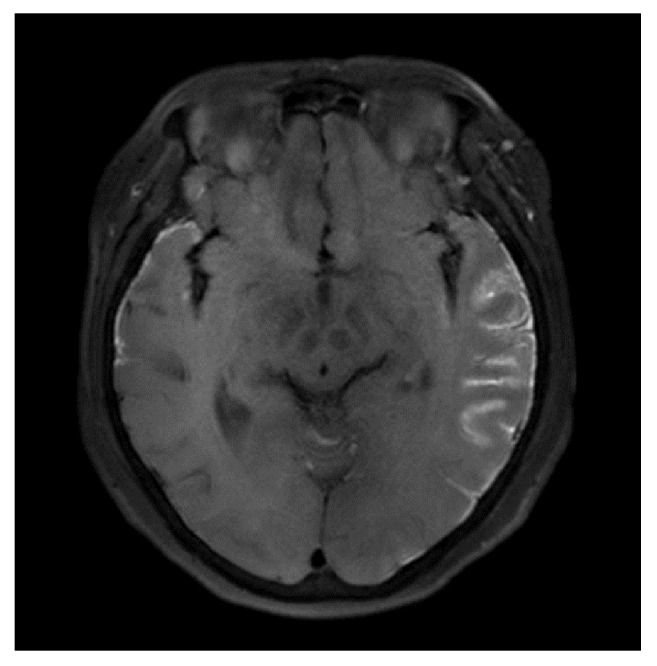
Cranial-enhanced MRI T1-weighted images reveal abnormal linear hyperintensity mainly on the left lateral lobe.

A 55-year-old Japanese woman, gravida 2 para 2, underwent surgery for a tumor in the left ovary (11×13×12 cm). Laparotomy revealed the swelling of both ovaries, rectum involvement, and peritoneal dissemination from the pelvic cavity to the upper abdominal cavity. She underwent a hysterectomy, bilateral salpingo-oophorectomy, omentectomy, and low-anterior resection of the rectum, which resulted in suboptimal surgery. Histological diagnosis confirmed high-grade serous carcinoma in the adnexal mass and peritoneal biopsy. She was diagnosed as stage IIIC ovarian cancer according to the International Federation of Gynecology and Obstetrics (FIGO) classification. Six cycles of adjuvant chemotherapy combining paclitaxel (180 mg/m^2^) and carboplatin (area under the curve (AUC) = 5) were administrated every 3 weeks, and her serum levels of CA125 decreased to normal. Thirteen months after the end of therapy, the same regimen plus bevacizumab was added because relapse sites were confirmed in the pelvis and also her CA-125 levels were elevated. After three cycles of chemotherapy, the regimen was changed to doxorubicin (60 mg/m^2^) because of progressive disease. She developed dizziness, back pain, and severe headaches without neurologic signs after two cycles of the therapy, at forty-three months after the primary surgery. After hospitalization, physical and neurological examination showed normal results and no parenchymal lesion was detected on a contrast-enhanced CT scan of the cranium. Diagnostic lumbar puncture was performed the next day, which revealed carcinomatous cells of ovary origin in the cerebrospinal fluid (CSF) (Figure 1). Magnetic resonance imaging (MRI) demonstrated abnormal hyperintensity in the cerebral sulci, mainly in the left lateral, occipital lobes, and folia in the cerebellar hemispheres and vermis on FLAIR with enhancement after gadolinium injection (Figure 2). On the basis of these results, she was diagnosed with carcinomatous meningitis. High-dose corticosteroid therapy was begun, although systemic or intrathecal chemotherapy was not added because of her poor performance status. Her general condition and consciousness declined rapidly and she died forty-two days after the diagnosis. An autopsy was not performed.

**Figure 3:**
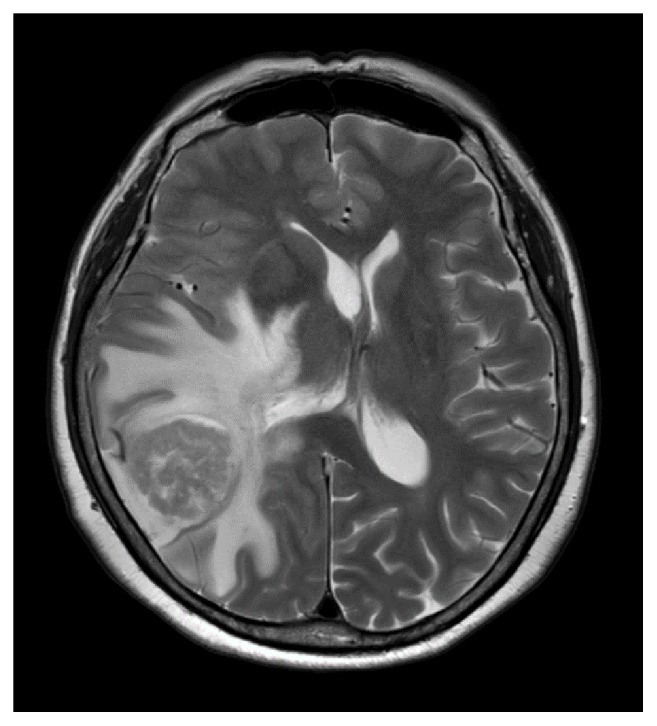
Axial T2-weighted brain MRI scan shows a solitary metastatic lesion in the right temporal lobe with brain edema.

The second case was a 63-year-old Japanese woman, gravida 2 para 2, with a history of hysterectomy because of fibroids at 45 years of age. A partially solid cystic tumor (5×4×6 cm) was detected in the pelvis at a private clinic. She was referred to our hospital for surgery. Her serum CA125 and CA19-9 levels were not elevated before surgery. Laparotomy revealed a tumor in the left ovary, and she was diagnosed as adenocarcinoma by frozen section without peritoneal lesions. The capsule of the tumor was intact and ascites was scanty. We performed bilateral salpingo-oophorectomy and omentectomy, resulting in compete surgery. Cytology of ascites was negative and the final pathological examination showed mucinous carcinoma limited to the left ovary (FIGO stage IA). After the surgery, she was followed up every 3–6 months at the outpatient department. After thirty-eight uneventful months, she developed headaches, dizziness, memory problems, and weakness of her left arm. A solitary tumor was found in the right lateral lobe (diameter 3 cm) on cranial MRI and a suspected relapse of the ovarian carcinoma was determined (Figure 3). She underwent surgical resection of the cerebral lesion and pathological examination confirmed metastatic disease. Gamma-knife radiosurgery followed (49 Gy) without further chemotherapy. Four months after the brain surgery, she presented with nausea and vomiting caused by ileus. A CT scan revealed multiple recurrent tumors in the pelvic cavity. She underwent laparotomy, but we could not resect these multiple recurrent lesions, and colectomy was done to improve ileus. Postoperatively, we used chemotherapy combining paclitaxel (180 mg/m^2^) and carboplatin (AUC=5). Despite the therapy, she was diagnosed as a progressive disease after three cycles. At the same time, her performance status declined gradually without intracranial relapse and neurological symptoms. She succumbed to abdominal involvement nine months after resection of the cerebral lesion and no autopsy was performed.

## 3. Discussion

Herein, we report two rare cases of CNS metastases from ovarian carcinoma. The first case was leptomeningeal metastases with no obvious parenchymal lesions, and the second case was solitary brain metastases with neurological symptoms as the first manifestation of relapse in the cerebrum regardless of her early clinical staging. CNS metastases observed in stages III and IV account for about 86% of cases whereas stage I accounts for only 9–10% [2, 3].

Brain metastases from the lung, breast, renal, and colorectal carcinoma and melanoma are common and occur in up to 40% of cases [4, 5], whereas leptomeningeal metastases are commonly seen in melanoma, lung, and breast carcinoma in 3–5% of cases [6]. The incidence of metastases from epithelial ovarian carcinoma to the brain parenchyma and leptomeninges is 0.9–3.3% [7–11] and 0.06% [12], respectively, indicating their rarity. Recent studies reported an increase in the incidence of metastases during the past decade. Chiang et al. reported a single institutional study where the incidence of metastases was increased from 0.18% (1995–1999) to 2.12% (2005–2009) [3]. The main reasons for this increase might be improvements in brain imaging and treatment for local relapse.

Almost 90% of CNS metastases develop an average of 25 months after the initial treatment [2]. Nasu et al. recently reported that CNS metastases from ovarian carcinoma had a statistically better prognosis than those from corpus and cervical carcinoma [2]. The median survival time was 12.5 months for ovarian cancer and 6.2 and 4.8 months for corpus and cervical cancer, respectively. Therefore, despite its poor prognosis, a prompt diagnosis of CNS metastases might provide an opportunity for appropriate treatment and palliative care.

The most common site of metastasis in the CNS is the cerebral hemisphere (75%) followed by the cerebellum (11%) and meninges (7.3%) [13]. Symptoms of CNS metastases from ovarian carcinoma were confirmed in approximately 90% of patients at diagnosis [2, 14]. A headache is the most common symptom seen in 40–50% of patients, which results from brain edema and/or hydrocephalus [15]. Other symptoms such as confusion, dizziness, decreased mental status, consciousness disturbance, general weakness, ataxia, and neurological motor deficits are also observed depending on the location of the metastases.

Symptoms of leptomeningeal metastases without parenchymal lesions are caused by increased intracranial pressure and meningeal irritation. The most common symptoms are headache and seizures. Gait difficulties from weakness or ataxia, memory problems, incontinence, and sensory abnormalities are sometimes reported, although classic cerebral signs such as hemiparesis and aphasia are less common. Cases with symptoms such as deafness, vertigo, facial weakness, and blindness as a first manifestation were also previously reported [16–18]. Both our cases showed headache and other neurological signs, while localized neurological signs were confirmed in the cerebral metastases case. Symptoms of cranial nerves were not observed in our cases.

Diagnosis of brain metastases is usually based on imaging techniques, but the diagnosis of carcinomatous meningitis can be difficult. The first step of the examination is a detailed check of neurological findings. After the physical examination, a computed tomography (CT) scan is used to detect metastatic lesions and rule out cerebrovascular events. MRI imaging is the most useful tool for the diagnosis of brain lesions as well as leptomeningeal metastases showing nodular subarachnoid enhancement after gadolinium injection. These findings were reported in 75–90% of patients whose cerebrospinal fluid (CSF) cytology was positive [19, 20]. The optimal test for a definitive diagnosis of leptomeningeal metastases is the detection of cancer cells in the CSF. However, the sensitivity of this method is low; for the first sample it is approximately 45%–75% [21, 22], although repetition of a sample three times can improve the sensitivity to 90% or more [22]. The sensitivities of MRI with gadolinium enhancement and enhanced CT scanning for the diagnosis of carcinomatous meningitis are approximately 70% and 30%, respectively [23–25]. Our first case was positive for MRI imaging and CSF examination at the first sampling. These findings allowed a prompt diagnosis, but also indicated severe meningitis. Serum CA125 levels are a useful biomarker to diagnose the relapse of epithelial ovarian carcinoma. However, it is of limited value for the detection of CNS metastases because it is impermeable to the blood-brain barrier. Anupol et al. reported that ten of 15 patients (66.6%) with brain metastases from ovarian carcinoma had elevated serum CA125 levels [26]. In our cases, the first case had already shown an elevated CA125 level because of other multiple relapses and the second case was negative for CA125 levels throughout the clinical course. Therefore, we were unable to use CA125 levels as a manifestation of brain metastases.

The mechanism of CNS metastases might involve vasculopathy or direct vascular obliteration by cancer cells, while that of leptomeningeal metastases remains the subject of investigation. It is suspected that cancer cells may reach the leptomeninges through hematogenous spread, direct invasion from parenchymal lesions, or spread along the perineurium from cranial or spinal nerves. After the cancer cells reach the subarachnoid space, they spread to the meninges directly through the CSF. Our leptomeningeal metastases case had multiple intra-abdominal relapses mainly in the pelvis with no evidence of liver, lung, or parenchymal lesions. We speculate the path of spread might be from spinal cord lesions. Although she had back pain, indicating spinal symptoms, we did not perform MRI of the spine.

The type and amount of CNS metastases were 42% for single lesions, 55% for multiple parenchymal lesions, and 3% for leptomeningeal disease [27]. To date, no clinical trials have been initiated for CNS metastases from ovarian carcinoma because of its rarity. Surgical resection, whole-brain radiotherapy, systemic chemotherapy, and gamma knife stereotactic radiosurgery alone or in combination have been used, dependent on the patient's condition and the metastatic sites in the brain. In a review article, Piura et al. reported that a significantly better survival was observed for multimodal therapy compared with whole-brain radiotherapy alone [27]. Cohen et al. also reported that surgical resection of solitary ovarian cancer metastases followed by whole-brain radiotherapy resulted in better survival compared with radiotherapy alone [7]. A limited meta-analysis recommended surgical resection followed by radiation therapy for patients with a solitary lesion and well-controlled relapse sites. In our case, cranial surgery followed by radiation therapy was performed because of the solitary lesion. Her neurological symptoms were improved remarkably even after the pelvic relapses were confirmed. These therapies were effective at improving her neurological symptoms without adverse effects.

The goal of treatment for leptomeningeal metastases is mainly the palliation of symptoms. The median interval between initial diagnosis and detection of leptomeningeal metastases was 26 months and its prognosis is extremely poor with a median survival ranging from 1.8 to 5.7 months [28]. In a clinical review, 67% of patients died within two months after the diagnosis [29]. Treatments are usually based on experiences with other carcinomas, because of its rarity. Chemotherapy and radiotherapy have been used; most cases were treated with intrathecal chemotherapy using methotrexate, with or without whole-brain radiation therapy. In some reports, treatment with intrathecal methotrexate succeeded in prolonging the survival period from 6 to 18 months [30–33]. Regarding our case, we had no chance to treat with methotrexate for palliation as her condition declined rapidly after the diagnosis.

## 4. Conclusion

In conclusion, CNS metastases from epithelial ovarian carcinoma have become a critical issue for clinicians, especially in patients receiving long-term chemotherapy. Currently, routine brain imaging is not recommended during the follow-up period for epithelial ovarian carcinoma. Clinicians should consider metastases to the CNS whenever a cancer patient shows neurological symptoms or behavioral changes. If a prompt diagnosis is made, it might lead to a better prognosis and the early initiation of palliative care, which should be beneficial.
